# CMW-Net: an adaptive robust algorithm for sample selection and label correction

**DOI:** 10.1093/nsr/nwad084

**Published:** 2023-03-25

**Authors:** Jun Shu, Xiang Yuan, Deyu Meng

**Affiliations:** School of Mathematics and Statistics, Xi’an Jiaotong University, China; Ministry of Education Key Lab of Intelligent Networks and Network Security, Xi’an Jiaotong University, China; School of Mathematics and Statistics, Xi’an Jiaotong University, China; Ministry of Education Key Lab of Intelligent Networks and Network Security, Xi’an Jiaotong University, China; School of Mathematics and Statistics, Xi’an Jiaotong University, China; Ministry of Education Key Lab of Intelligent Networks and Network Security, Xi’an Jiaotong University, China; Peng Cheng Laboratory, China; Pazhou Laboratory (Huangpu), China

## Abstract

A class-aware sample weighting algorithm is developed for general label noise problems. The algorithm can effectively tackle complicated and diverse noisy label tasks, winning the Championship of the ‘Arena Contest’ Track 1 of 2022 Greater BayArea (Huangpu) International Algorithm Case Competition.

## PROBLEM

Deep neural networks (DNNs) have recently achieved impressive performance in various applications. Such success is largely attributed to massive but carefully labeled data expected to properly and sufficiently simulate testing environments. However, the data collection and annotation processes are often expensive and time consuming, making obtained training datasets in real-life applications error prone, especially with data bias from ideal testing distributions. One typical form of such data bias issues is label noise. Specifically, it has been demonstrated that DNNs can easily overfit to noisy labels [1], which eventually results in their poor generalizability in test cases. Hence, achieving a good generalization capability in the presence of noisy labels is a key challenge for deep learning.

Sample selection and label correction are two of the most commonly used techniques in dealing with noisy labels. Sample selection aims to identify relatively faithfully labeled examples from noisy training data, and then amplify their roles, meanwhile suppressing those noisy ones in the training process. Label correction needs to identify wrongly labeled examples and then possibly correct their labels for re-introduction into training. Recently, various sample selection and label correction strategies have been proposed. While these methods are delicately designed for specific tasks, most of them are hardly usable for general and diverse noisy label issues, especially those real-life heterogeneous label noise tasks, which are notoriously complicated since noisy labels always have different inter-class noise structure forms, like noise rate, noise type and class capacity. It is thus critical and challenging to develop novel sample selection and label correction algorithms generally performable for varieties of noisy label situations and especially those real-life ones.

To promote the development of deep learning techniques in real-life applications, the 2022 International Algorithm Case Competition requires competitors to design a sample selection and label correction algorithm for dealing with the aforementioned heterogeneous and diverse label noise cases (see the Evaluation section), and achieving good performance on test datasets.

## ALGORITHM

### Motivation behind the algorithm design

The aim of sample selection and label correction could be naturally realized by solving the optimization problem


\begin{eqnarray*} && \min _{\boldsymbol {w}} \frac{1}{N} \sum _{i=1}^N v_i \ell (f_{\boldsymbol {w}}(\boldsymbol {x}_i),\hat{y}_i) \\ &&\quad +\, (1-v_i) \ell (f_{\boldsymbol {w}}(\boldsymbol {x}_i),z_i), \end{eqnarray*}

where *v_i_* ∈ [0, 1] denotes sample weight, indicating the extent to which sample $(\boldsymbol {x}_i,\hat{y}_i)$ functions in training. If *v_i_* → 1 then $\hat{y}_i$ is probably a clean label and the model tends to use it for training; otherwise, if *v_i_* → 0 then $\hat{y}_i$ is probably noisy and the model tends to use pseudo soft label *z_i_* generated by classifier $f_{\boldsymbol {w}}$ as the corrected label for training. We expect to recover the underlying clean label of each sample *x_i_* if we assign proper *v_i_* to it. Accordingly, the key to designing the expected algorithm is to develop a proper sample weight-assigning principle for general label noise problems, so as to achieve the desired sample selection and label correction.

There exist many strategies to deal with the aforementioned issue [2] that achieve fine performance on specific problems. The competition scene, however, is more challenging for current algorithms. As required by competition, the label noise cases involved in all competition tasks exhibit several typical characteristics: complex and diverse label noise forms, heterogeneous and varied noise structures among classes, multiple data modalities, massive data size and also other forms of data biases like class imbalance. Comparatively, the effectiveness of existing algorithms may decrease under the competition scene. To design such a consistently usable sample weighting framework with a unified form for all these tasks is evidently not an easy task and actually limited studies are currently available. This is the intrinsic purpose of setting this competition.

To achieve such an expected unified sample weighting strategy, our previous Meta-Weight-Net (MW-Net) method uses an explicit sample weighting mapping [3] for realizing such a robust learning algorithm, which is adaptable for different learning tasks in a meta-learning manner. The mapping is modeled as a standard multilayer perceptron (MLP) net, whose hidden node uses the rectified linear unit as the activation function with the output utilizing the sigmoid activation function [3,4]. By setting its input as the training loss of a sample and its output as the sample weight, the mapping is known with a strong fitting capability to represent a wide range of weighting function forms (see Fig. 1 of ref. [3] for an easy illustration). Such mapping can thus yield a rational weighting function directly from data in the homogeneous noisy labels as well as class imbalance cases, finely complying with common setting schemes in traditional methods for both data bias situations, and can also fit more complicated scenarios beyond manual sample weighting setting approaches.

To further enhance the ability of the MW-Net method for dealing with highly heterogeneous label noise as specified in this competition, our key insight is to adaptively distinguish the training data with heterogeneous bias characteristics into multiple clusters, each with relatively more homoscedastic label noise bias. To this end, compared to the MW-Net method only using the sample-level feature (i.e. loss) to distinguish the individual bias property of each sample, a supplementary task-level feature of training tasks (corresponding to all training classes) needs to be further employed to deliver their specific intra-class bias characteristic. This is beneficial to make the method capable of being aware of specific bias configurations of different training classes, accumulating tasks with approximately homoscedastic data bias to extract their targeted shared weighting schemes, while adaptively setting different weight mappings across task families with different bias cases (see Fig. 2 of ref. [5] for an easy illustration). Here we treat every class of training samples as a separate learning task; an illustration of this setting is discussed in ref. [5]. In this way, the challenging heterogeneous data bias issue is well handled, inspiring us to design a more advanced class-aware MW-Net (CMW-Net) method as follows.

### CMW-Net algorithm formulation

**Figure 1.**
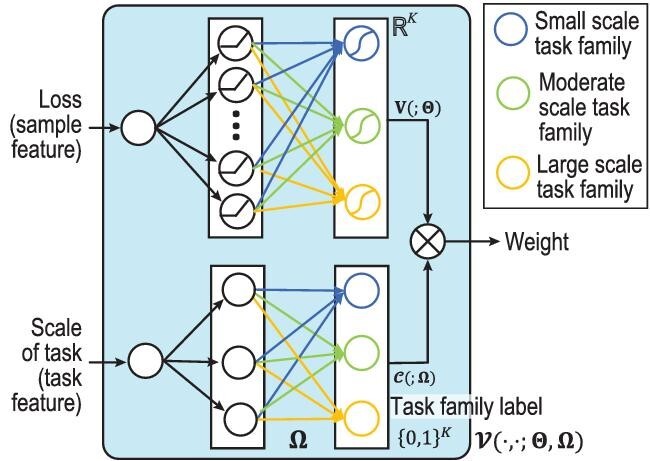
The architecture of the CMW-Net model. Adapted with permission from Shu *et al.* [5].

The CMW-Net algorithm, as shown in Fig. 1, aims to adaptively learn a proper weighting scheme for heterogeneous and diverse label noise in a meta-learning manner. It is formulated as


\begin{eqnarray*} \mathcal {V}(\ell _i,N_i;\Theta ,\Omega ) \!=\! {\mathbf {V}}(\ell _i;\Theta ) \!\otimes\! \mathcal {C}(N_i;\Omega ), \end{eqnarray*}

where $\mathcal {C}(N_i;\Omega ) \in \lbrace 0,1\rbrace ^K$ denotes the lower branch of the CMW-Net architecture (see Fig. 1), taking *N_i_* (the number of samples contained in the training class to which the sample *x_i_* belongs) as its input to represent the task feature, and ‘⊗’ denotes dot multiplication of two vectors. It includes a hidden layer containing *K* nodes, attached with *K* levels of scales $\Omega =\lbrace \mu _k\rbrace _{k=1}^{K}$ sorted in ascending order (i.e. μ_1_ < μ_2_ < ⋅⋅⋅ < μ_*K*_). In other words, we use the scale level of each training task (i.e. *N_i_*) to represent its task feature, and apply *K* means to obtain centers of clustering task families (i.e. $\Omega =\lbrace \mu _k\rbrace _{k=1}^{K}$). In our implementation, we set *K* = 3 and such a simple task feature is able to deliver helpful task patterns underlying the bias types of different training classes. More comprehensive task features will be further explored in our future research.

We denote by **V**(ℓ_*i*_; Θ) ∈ [0, 1]^*K*^ the upper branch of the CMW-Net architecture (see Fig. 1), built as an MLP net with sample loss (i.e. $\ell _i= \ell (f_{\boldsymbol {w}}(\boldsymbol {x}_i),\hat{y}_i)$) as its input, containing one hidden layer and a *K*-dimensional output. The *K* output weights correspond to the *K* different weighting schemes imposed on samples located on different task families.

We can optimize CMW-Net by solving the bi-level optimization problem [5]:


\begin{eqnarray*} && \lbrace {\Theta }^*,\Omega ^*\rbrace = \mathop {\arg \min }_{{\Theta ,\Omega }} \frac{1}{M} \sum _{i=1}^M L_i^{meta} \\ &&\qquad (\mathbf{w}^*(\Theta,\Omega)), \\ && \mathbf {w}^*(\Theta ,\Omega ) = \mathop {\arg \min }_{{\mathbf {w} }} \sum _{i=1}^N \\ &&\qquad \mathcal {V}(\ell _i,N_i;\Theta ,\Omega ) \ell (f_{\boldsymbol {w}}(\boldsymbol {x}_i),\hat{y}_i) \\ && +\, (1-\mathcal {V}(\ell _i,N_i;\Theta ,\Omega ))\ell (f_{\boldsymbol {w}}(\boldsymbol {x}_i),z_i), \end{eqnarray*}

where $L_i^{\mathrm{meta}}(\mathbf {w}) = \ell (f_{\mathbf {w}}(\boldsymbol {x}_i^{(\mathrm{meta})}), y_i^{(\mathrm{meta})})$ is the meta-loss computed on a pre-collected unbiased meta dataset $\mathcal {D}^{\mathrm{meta}} = \lbrace \boldsymbol {x}_i^{(\mathrm{meta})},y_i^{(\mathrm{meta})}\rbrace _{i=1}^M$, which represents the meta-knowledge of the unbiased sample-label distribution. See Section 3.4 of ref. [5] for algorithm details. In our implementation, we automatically generate a certain number of the most confident training samples in each iteration of the training process as meta-data, which is soundly motivated by the curriculum learning methodology [6,7], use a common semi-supervised learning technique [8] to produce pseudo label *z* and employ the finite difference approximation technique [9] to compute the second-order gradient efficiently. All implementations can be easily executed and reproduced by general users.

### EVALUATION

#### Tasks and datasets

The algorithms presented in the previous section are extensively tested with heterogeneous and diverse label noise datasets of the competition. Specifically, consider a classification problem with noisy labels $\hat{\mathcal {D}} = \lbrace (\boldsymbol {x}_i,\hat{y}_i)\rbrace _{i=1}^N$, where $\boldsymbol {x}_i \in \mathbb {R}^d$ denotes the *i*th training sample, $\hat{y}_i \in \lbrace 0,1\rbrace ^C$ is the one-hot encoding noisy label corresponding to $\boldsymbol {x}_i$, whose clean label is *y_i_*, and *N* is the size of the entire training dataset. The generative process of label noise can be formulated as


\begin{eqnarray*} p(\hat{y}| \boldsymbol {x}) &=& \sum _{i=1}^C p(\hat{y}| \boldsymbol {x}, y=i) \\ &&\times \, p(y=i| \boldsymbol {x}), \end{eqnarray*}

where $p(\hat{y}=j| \boldsymbol {x}, y=i)$ denotes the transition probability of a clean label *y* = *i* wrongly labeled as a noisy label $\hat{y} = j$ for sample $\boldsymbol {x}$, and satisfies $\sum _j p(\hat{y}=j| \boldsymbol {x}, y=i) =1$. To evaluate the capability of proposed algorithms against various label noises the preliminary competition sets the following four tasks.

**Task 1.** Assume that a clean label *y* = *i* is wrongly labeled as all other possible noisy labels $\hat{y} = j, j\ne i$ with the same probability, i.e.


\begin{eqnarray*} p(\hat{y} = j| \boldsymbol {x}, y=i) = \frac{\tau }{ C-1}, \end{eqnarray*}

where τ denotes the noise rate.

**Task 2.** Assume that a clean label *y* = *i* is wrongly labeled as ones in a label set $S_i \subset \{1,\cdots,C\}$, i.e.


\begin{eqnarray*} p(\hat{y} = j| \boldsymbol {x}, y=i) = \left\lbrace \begin{array}{@{}l@{\quad }l@{}}\tau _{ij}, & j \in S_i, \\ 0, & j \notin S_i, \end{array}\right. \end{eqnarray*}

where τ_*ij*_ is the probability of label *i* being wrongly labeled as *j*.

**Task 3.** Assume that a clean label *y* = *i* is wrongly labeled as a noisy label $\hat{y} = j$ with a probability dependent on sample $\boldsymbol {x}$, i.e.


\begin{eqnarray*} p(\hat{y}=j| \boldsymbol {x}, y=i) = \tau _{ij}(\boldsymbol {x})\quad\!\!\! \text{for all } i,\! j, \end{eqnarray*}

where $\tau _{ij}(\boldsymbol {x})$ is instance-dependent probability.

**Task 4.** Train DNNs with noisy labeled data in real-life scenes.

To further evaluate the capability of competing algorithms against general and extensive real-life label noise problems, the final competition sets the following two tasks, and only provides about six days to complete all tasks.

**Task 5.** Train DNNs with extensive desensitized noisy data (including 106 datasets), without access to raw data and noise structures.

**Task 6.** Train DNNs with a large-scale real-life noisy dataset.

The evaluation metrics comprise two aspects: (1) performance, including correction accuracy of noisy labels and accuracy on the test datasets; (2) scientificity, including universality, reproducibility, feasibility and efficiency in time and memory. For a detailed introduction on evaluation metrics and competition datasets, see https://www.cvmart.net/race/10343/des.

#### Performance of the CMW-Net model

*Learning adaptive weighting schemes for different data bias forms.* The CMW-Net model is applied to generally achieve proper weighting schemes for different data bias forms, including class imbalance, synthetic class-independent, class-dependent and feature-dependent label noise with different noise rates and training classes, and especially real-life heterogeneous data biases. See Figs 3 and 8 of ref. [5] for a better illustration.

*Achieving SOTA performance on diverse data bias issues.* The CMW-Net model is model agnostic, and is applied to help improve the performance of sample selection and label correction in a series of data bias issues, including datasets with class imbalance, different synthetic label noise forms and real-life complicated biased datasets, like Animal-10N, WebVision, WebFG-496, etc. This naturally leads to its competitive performance in Tasks 1–4 of this competition. Such an elaborately designed weighting strategy can also improve other robust learning tasks, like partial-label learning, semi-supervised learning and selective classification [5]. This capability means that it possesses good performance in dealing with Task 5 of this competition. Note that the CMW-Net model could benefit from the latest state-of-the-art (SOTA) methods and the performance of our method could be further boosted.

*Task-transferable weighting scheme.* The meta-learned weighting scheme mentioned above can be used in a plug-and-play manner, and directly deployed on unseen datasets, without needing to specifically tune extra hyperparameters of the CMW-Net algorithm. We have attempted to transfer the meta-learned weighting scheme obtained from a relatively small dataset (CIFAR-10) to a significantly larger-scale dataset (e.g. WebVision), and achieved superior results even beyond the SOTA methods specifically designed for the dataset (see Section 6 of ref. [5]). This function is especially meaningful for large-scale problems, like Task 6 in this competition. In particular, under the required short submission period during the competition, such a task-transferability function of the CMW-Net algorithm largely enhances the implementation efficiency of our algorithm by avoiding the extra time-consuming weighting function tuning process for handling such a large-scale problem.

*Solid theoretical guarantee.* We have established the corresponding statistical learning theory of such a task-transferable methodology learning manner. We refer the reader to ref. [4] for more details, which provides solid theoretical support for the effectiveness of our algorithm.

*Computational efficiency in time and memory.* In terms of computational cost in time and memory, the cost of the CMW-Net algorithm is substantially less than the requirements of the competition. This implies that our algorithm is potentially applicable for real-life applications, especially those large-scale ones with complicated data biases.

*Easy to implement.* Our algorithm is relatively easy to implement in general data bias cases. Its core code is less than 30 lines. We have made the completed code available at https://github.com/xjtushujun/CMW-Net so that it can be reproduced and applied to other complicated real-life problems by general users.

Our CMW-Net algorithm has been extensively tested with complex and diverse noisy label tasks in competition, especially massive noisy label datasets with unknown noise structures in Task 5, and a large-scale real-life biased dataset in Task 6, and achieved the best evaluation performance and oral defense score of the Competition, showing its potential usefulness for more practical datasets and tasks.

## FUTURE RESEARCH DIRECTIONS

After a careful investigation and analysis of the competition results, possible improvements and suggestions for future research are as follows.

*Learning with more data bias forms.* The competition mainly focuses on one typical data bias, label noise. The real-life scenarios may contain more data bias cases, e.g. class imbalance, joint sample-label noise and other weakly annotated forms like partial/complementary label annotation, multi-label data with only single-label annotation, semi-/barely supervised annotation, few-shot annotation. Besides, most real datasets are collected uncontrolled, inevitably resulting in dataset biases, e.g. out of distribution, spurious correlations, fairness, etc. It calls for more research attention on tackling these challenging issues. In particular, real-life large-scale datasets often encounter data privacy issues and high resource costs. Hence, designing computation-efficient and privacy-preserving algorithms could be another attractive future topic. Plug-and-play weighting schemes could provide a new research perspective on this topic.

*Machine learning for data.* The competition makes efforts to design algorithms for cleaning and annotating data used to train DNNs. In fact, many current deep learning algorithms require training data to be of high quality. The data need to be manually cleaned, valued and annotated beforehand, but not adaptively designed, generated and rectified by algorithms. It, however, is always notoriously costly and difficult to create high-quality datasets. Hence, when applying deep learning techniques to real-life applications, it is meaningful to develop algorithms to eval-uate, synthesize, clean and re-annotate the low-quality data to possibly improve their quality for supporting trustworthy DNNs training. Such data-centric machine learning provides a critical and prospective research path to mitigate bias and improve the generalizability and reliability of the DNNs.

*Adapting to dynamically changing tasks.* The competition contains significantly diverse label noise tasks, and requires the use of a unified algorithm to tackle all tasks. Considering the dynamic evolution insight of real-life environments, developing algorithms adapting to dynamically changing tasks with variant data biases should be an essential topic that deserves more research attention. In particular, when acquiring and using data continuously, it is worth investigating how to automatically select data that could more faithfully reflect the real testing environments, sending that data to expert annotators for retraining DNNs models.

*Simulating learning methodology.* Inspired by this weighting-function-imposing methodology learning [5] for the competition, a critical extension is to simulate the methodology of how to set other hyperparameters in machine learning, e.g. learning rate [10], pseudo label [11], loss function [12], etc. This new learning manner provides valuable insights for machine learning automation and meta-learning [4].

*Novel transfer learning theory.* Current works mainly study the theoretical guarantee for transfer learning via representation learning. Comparatively, the task-transferable weighting scheme provides a new viewpoint to understand transfer learning. Specifically, this process can be regarded as extracting the common hyperparameter setting policy (representation learning could be a special case) shared by diverse tasks, and then transferring this learned policy to new query tasks [4]. The theory of such transfer learning could provide more macroscopical and essential task generalization toward real dynamic environments.
